# Changing Patterns of Cutaneous Melanoma Mortality in Brazil: A Nationwide Time-Series Analysis (2000-2024)

**DOI:** 10.7759/cureus.114446

**Published:** 2026-08-12

**Authors:** Maria Eduarda B Ribeiro, Marcella S Abizaid, Maria Eduarda K Garcia, Thaís L Froes, Lara S Tasca, Sofia G Calil Lopes, Mariana M Rizzolli, Bettina A e Souza Bichara, Leticia P Scaramussa, Humberto G Moreira

**Affiliations:** 1 Faculty of Medicine, Universidade do Oeste de Santa Catarina, Florianopolis, BRA; 2 Faculty of Medicine, Universidade de Santo Amaro, Sao Paulo, BRA; 3 Faculty of Medicine, Centro Universitário Souza Marques (UniSM), Rio de Janeiro, BRA; 4 Faculty of Medicine, Anhembi Morumbi University, Sao Paulo, BRA; 5 Faculty of Medicine, Universidade Nove de Julho, Sao Paulo, BRA; 6 Faculty of Medicine, Universidade de Marília, Marília, BRA; 7 Faculty of Medicine, Universidade de Caxias do Sul, Caxias do Sul, BRA; 8 Faculty of Medicine, Universidade do Grande Rio (Unigranrio), Rio de Janeiro, BRA; 9 School of Medicine, Federal University of Goiás, Goiânia, BRA; 10 Academic Research Organization, Einstein Hospital Israelita, Sao Paulo, BRA

**Keywords:** cutaneous malignant, melanoma, mortality, mortality in melanoma, mortality registries, mortality trends in brazil

## Abstract

Background: Cutaneous melanoma accounts for most deaths from skin cancer, and mortality is consistently higher in men than in women. National trends in Brazil have not been characterized using population estimates rebased to the 2022 Census. Materials and methods: We conducted a nationwide ecological time-series study of deaths from cutaneous melanoma (ICD-10 C43) recorded in Brazil’s Mortality Information System from 2000 to 2024. Population denominators were obtained from the 2024 revision of the Brazilian Institute of Geography and Statistics projections, rebased to the 2022 Census. Rates were directly age-standardized to the 2022 Brazilian population; 95% confidence intervals (CIs) were estimated using the Fay-Feuer gamma method, a standard approach for deriving exact confidence limits for standardized rates from count data. National and subgroup trends were assessed using segmented log-linear regression, which identifies the calendar years in which a trend changes direction. Differences by sex, region, and age group were examined using age-adjusted negative binomial models with population offsets, which compare death rates between groups of different population sizes. The sex interaction was prespecified as a secondary, exploratory comparison and was not adjusted for multiple testing. The COVID-19 period was assessed using an interrupted time-series model with 2020 specified a priori. Results: Between 2000 and 2024, 38,346 deaths were attributed to cutaneous melanoma. Annual deaths increased from 1,027 to 2,031, whereas the age-standardized rate was essentially unchanged, from 0.91 to 0.92 per 100,000 population. Mortality increased by 0.83% per year (95% CI 0.52 to 1.14) until 2016 and declined by 1.12% per year (−1.69 to −0.54) thereafter. The full-period average annual percentage change (AAPC) was 0.17% (−0.01 to 0.36; p = 0.065). Men had higher rates throughout. Sex-specific segmented models showed an increase followed by a decline in men and an overall stable trend in women. An exploratory, non-multiplicity-adjusted interaction offered limited and inconsistent evidence of a more favorable trajectory in women than in men (p = 0.037). Mortality declined in all groups younger than 60 years and increased overall among adults aged 60 years or older, although the rise leveled off after 2015. The Southeast declined, the South was stable overall, and the North and Northeast increased from very low initial rates. The pandemic was associated with a one-time 6.60% downward shift in mortality in 2020, without a change in slope. Using population estimates not rebased to the 2022 Census changed the national AAPC from 0.17% (−0.01 to 0.36) to −0.39% (−0.60 to −0.18). Conclusions: The increasing number of melanoma deaths in Brazil was driven largely by population growth and aging rather than by a sustained increase in age-standardized mortality. A national decline began around 2016, but trends remained heterogeneous by age and region. The material change in the national AAPC after revision of the population denominators demonstrates that denominator vintage is not merely a technical consideration in long-term mortality studies but can materially affect epidemiological conclusions.

## Introduction

Cutaneous melanoma accounts for a small proportion of skin cancers but is responsible for most skin-cancer deaths, reflecting its capacity for early metastatic spread once tumor thickness increases [1]. Although incidence has risen in many fair-skinned populations, mortality has followed a more heterogeneous course, declining in several high-income countries while remaining stable or increasing elsewhere [2,3]. These differences have been linked to prevention, stage at diagnosis, tumor characteristics, and access to effective systemic therapy [2-4].

Men experience consistently higher melanoma mortality and poorer survival than women [4]. Differences in stage, anatomical site, and age explain only part of this disadvantage, supporting national analyses that examine both mortality level and temporal trajectory rather than assuming a uniform sex effect across populations [3,4].

Brazil provides a distinctive setting for this analysis. Its continental size, demographic admixture, regional variation in constitutive skin phototype, and unequal distribution of diagnostic and oncology services create pronounced heterogeneity in melanoma risk and outcomes [5,6]. Earlier Brazilian studies reported increasing incidence, higher mortality in men, and marked geographical variation, but most covered earlier periods or individual cities or states, or used population denominators that preceded the 2022 Census revision [7-9].

Two developments warrant an updated national assessment. First, recent advances in melanoma diagnosis and treatment occurred after the observation windows of most Brazilian studies [9]. Second, the COVID-19 pandemic disrupted skin-cancer diagnosis and treatment, creating a possible short-term disturbance in mortality records [10,11]. The present analysis extends beyond earlier Brazilian series in three respects: (1) a longer and more recent observation window, to 2024; (2) population denominators rebased to the 2022 Census rather than the earlier projection series used previously; and (3) formal incorporation of the COVID-19 period through a prespecified interrupted time-series model. A comparable approach was recently applied to state-level mortality in the United States [12], illustrating how denominator revision and extended follow-up can alter national conclusions. We therefore examined national trends in cutaneous melanoma mortality in Brazil from 2000 to 2024, with analyses by sex, age, and macro-region.

## Materials and methods

Study design and setting

We performed a nationwide, population-based ecological time-series study of cutaneous melanoma mortality in Brazil from 1 January 2000 to 31 December 2024. Brazil comprises 26 states and the Federal District, grouped into five macro-regions: North, Northeast, Central-West, Southeast, and South. The unit of analysis was the annual population aggregate, stratified by sex, age group, and macro-region. All descriptive, segmented regression, interaction, interrupted time series, and sensitivity analyses used the same 2000-2024 observation window.

Data sources

Death records were obtained from the Mortality Information System (Sistema de Informações sobre Mortalidade, SIM) of the Brazilian Ministry of Health through DATASUS/TabNet. We included deaths for which malignant melanoma of the skin (International Classification of Diseases, 10th Revision, code C43) was recorded as the underlying cause, tabulated by year of death, sex, age group, and macro-region. The year of death, rather than the year of registration, was used as the temporal variable. Data were extracted on 1 July 2026 from the final consolidated SIM release for 2024, updated by the Ministry of Health on 2 December 2025.

Annual resident population denominators by sex, five-year age group, and macro-region were obtained from the Brazilian Institute of Geography and Statistics (IBGE) [13]. Full population counts by year, sex, age group, and macro-region are publicly available at this source. This series incorporates the 2022 Census and its post-enumeration survey. The rebased series was used consistently for every year of the study, and national denominators were obtained as the sum of the five regional populations. The effect of using the earlier, pre-Census projection series was examined in a prespecified sensitivity analysis.

Outcomes and standardization

The primary outcome was the age-standardized mortality rate from cutaneous melanoma per 100,000 person-years. Crude rates were calculated using annual deaths and the corresponding resident population. Rates were directly standardized to the 2022 Brazilian population from the same rebased IBGE series across 17 age groups, ranging from 1-4 years to ≥80 years. A common standard population was applied to every year, sex, and macro-region. Deaths and population younger than one year were consistently excluded from the numerator and the denominator. Ninety-five percent confidence intervals (CIs) for age-standardized rates were calculated using the gamma method of Fay-Feuer.

For age-specific analyses, we used within-band mortality rates without standardization. Regressions were weighted by the annual number of deaths in each band, and years with zero deaths were excluded from the log-rate regression for that band.

Statistical analysis

Temporal trends were estimated using weighted segmented log-linear regression fitted to annual mortality rates on the log scale, with inverse-variance weights derived by the delta method. A maximum of two joinpoints and a minimum of five annual observations per segment were prespecified. The number of joinpoints was selected by sequential permutation testing with 4,499 permutations, and 95% CIs for joinpoint locations were obtained by parametric bootstrap with 2,000 replicates. Annual percentage changes (APCs) and average annual percentage changes (AAPCs) were derived from the fitted log-slopes and their covariance matrix [14].

Across all comparisons, the segmented log-linear models constituted the primary trend analysis. The negative binomial interaction models were secondary and exploratory and were used to formally test between-group differences rather than to re-estimate trends. Differences in temporal trends by sex, macro-region, and age group were tested in separate negative binomial regression models with a log link, annual deaths as the outcome, log resident population as an offset, and fixed effects for five-year age groups. Each model included year, group, and year-by-group interaction terms. Women were the reference category in the sex model, the Southeast in the regional model, and the youngest group in the age-group model. The national segmented model was the primary trend analysis. Sex-specific segmented models were descriptive, whereas the year-by-sex interaction was prespecified as a secondary exploratory analysis and interpreted according to effect size, CI, and consistency with the segmented estimates.

The COVID-19 period was assessed using an interrupted time-series model fitted to the same national age-standardized series and weights, with 2020 specified a priori as the interruption point. The model estimated the pre-pandemic slope, an immediate level change, and a post-pandemic change in slope. Because the segmented model estimated the timing of inflection from the data, it was designated as the primary model; the interrupted time-series analysis was complementary. Model fit was compared using the Akaike information criterion.

Four sensitivity analyses were prespecified: exclusion of 2024; exclusion of 2020-2022; repetition of the national and sex-specific analyses using the pre-Census population series; and separate linear trends for 2000-2019 and 2020-2024. Statistical tests were two-sided, with p < 0.05 considered statistically significant. Analyses were performed using R (version 4.6.1; R Foundation for Statistical Computing, Vienna, Austria) [15].

Ethical considerations

The study used only aggregated, publicly available data without individual identifiers. Under Brazilian National Health Council Resolution 510/2016, research using aggregated public-domain data is exempt from review by a Research Ethics Committee.

## Results

Deaths and mortality rates

Between 2000 and 2024, 38,346 deaths were attributed to cutaneous melanoma in Brazil. Annual deaths increased from 1,027 in 2000 to 2,031 in 2024, while the crude mortality rate rose from 0.60 to 0.97 per 100,000 population. By contrast, the age-standardized mortality rate remained virtually unchanged, at 0.91 per 100,000 (95% CI 0.85-0.97) in 2000 and 0.92 (0.88-0.96) in 2024 (Table 1).

**Table 1:** Deaths and mortality rates due to melanoma in Brazil and its geographical regions (2000 and 2024) * Rates per 100,000 population. Age-standardized rates were directly standardized to the age distribution of the 2022 Brazilian population using IBGE population projections (2024 revision), rebased to the 2022 Census; 95% CIs were calculated using the Fay-Feuer gamma method. The same denominator series was used for all years [13]. CI: confidence interval, IBGE: Brazilian Institute of Geography and Statistics

Area	Deaths, 2000	Crude rate, 2000*	Age-standardized rate, 2000* (95% CI)	Deaths, 2024	Crude rate, 2024*	Age-standardized rate, 2024* (95% CI)
Brazil - overall	1,027	0.6	0.91 (0.85-0.97)	2,031	0.97	0.92 (0.88-0.96)
Brazil - men	566	0.67	1.00 (0.92-1.09)	1,160	1.13	1.08 (1.02-1.14)
Brazil - women	461	0.53	0.82 (0.74-0.90)	871	0.81	0.77 (0.72-0.82)
North	7	0.05	0.10 (0.03-0.22)	70	0.38	0.52 (0.40-0.65)
Northeast	80	0.17	0.27 (0.21-0.33)	324	0.57	0.58 (0.51-0.64)
Southeast	559	0.76	1.08 (0.99-1.18)	872	0.99	0.87 (0.82-0.93)
South	337	1.34	1.90 (1.70-2.12)	641	2.08	1.82 (1.69-1.97)
Central-West	44	0.37	0.65 (0.46-0.89)	124	0.74	0.81 (0.67-0.96)

Men accounted for 566 (55.1%) deaths in 2000 and 1,160 (57.1%) in 2024. Age-standardized mortality was higher in men than in women at both time points: 1.00 per 100,000 (95% CI 0.92-1.09) versus 0.82 (0.74-0.90) in 2000 and 1.08 (1.02-1.14) versus 0.77 (0.72-0.82) in 2024.

National temporal trends

**Figure 1:**
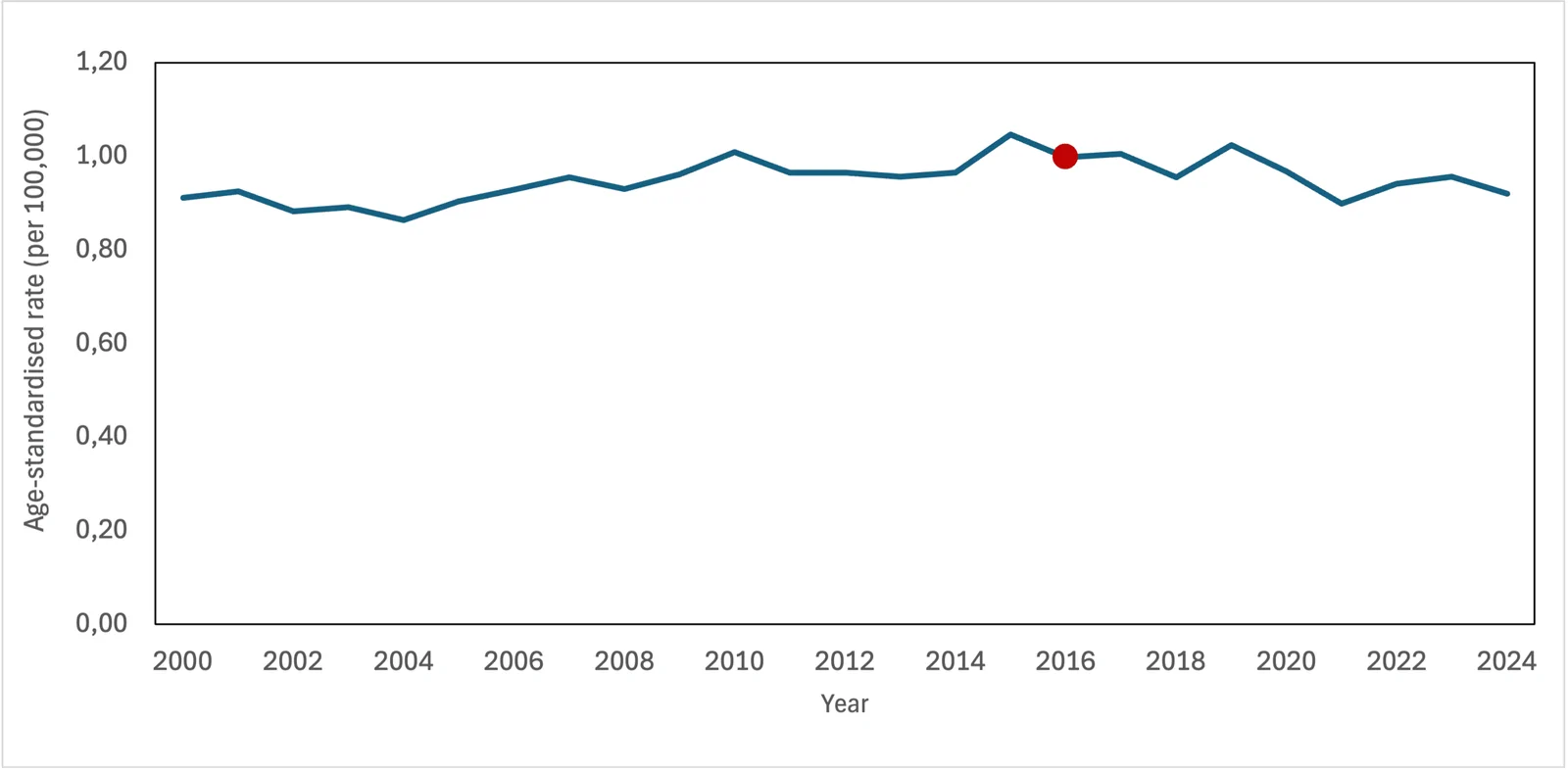
National age-standardized mortality rate for melanoma with joinpoint segments indicated (2000-2024) Deaths were obtained from the Mortality Information System and population denominators from the 2024 revision of the IBGE projections, rebased to the 2022 Census. Rates were directly standardized to the 2022 Brazilian population. The solid line shows the observed annual rates and the red marker identifies the estimated joinpoint in 2016. Mortality increased by 0.83% per year from 2000 to 2016 and declined by 1.12% per year thereafter. IBGE: Brazilian Institute of Geography and Statistics

Segmented regression identified one inflection point in 2016 (95% CI 2012-2019) (Figure 1). Age-standardized mortality increased by 0.83% per year (95% CI 0.52-1.14; p < 0.0001) between 2000 and 2016 and declined by 1.12% per year (−1.69 to −0.54; p = 0.0006) thereafter. Over the full study period, the AAPC was 0.17% (−0.01 to 0.36; p = 0.065), indicating no significant overall trend.

Regional trends

Age-standardized mortality differed markedly across macro-regions (Table 1). In 2024, rates were highest in the South (1.82 per 100,000, 95% CI 1.69-1.97), followed by the Southeast (0.87, 0.82-0.93), Central-West (0.81, 0.67-0.96), Northeast (0.58, 0.51-0.64), and North (0.52, 0.40-0.65).

**Figure 2:**
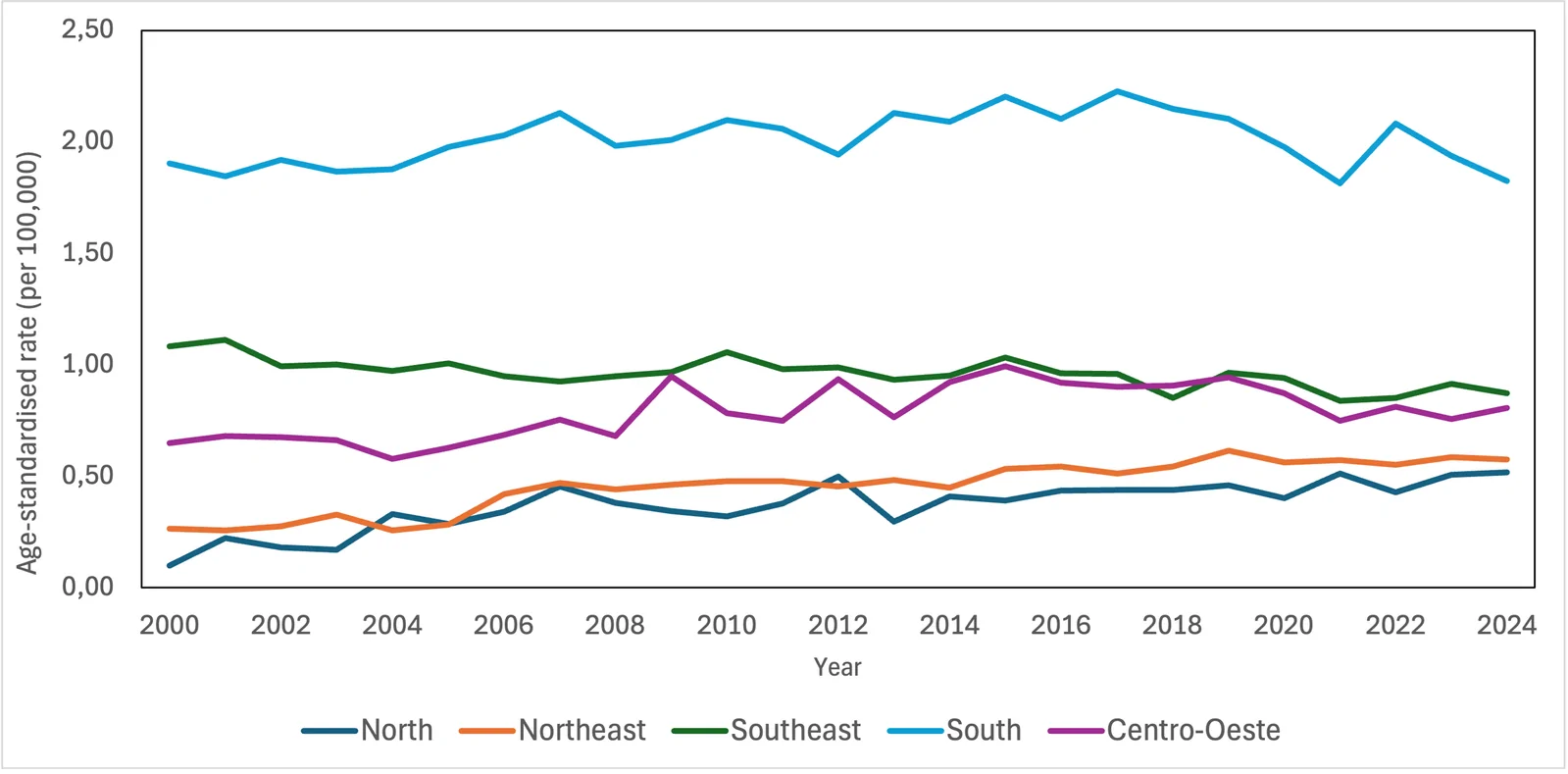
Age-standardized mortality rate by region (2000-2024) Deaths were obtained from the Mortality Information System and population denominators from the 2024 revision of the IBGE projections, rebased to the 2022 Census. Rates were directly standardized to the 2022 Brazilian population. Lines show annual observed rates for the North, Northeast, Southeast, South, and Central-West regions. Regional trends were estimated using segmented log-linear regression and compared formally using age-adjusted negative binomial regression with population offsets. IBGE: Brazilian Institute of Geography and Statistics

Temporal patterns were heterogeneous (Figure 2). Mortality declined continuously in the Southeast (AAPC −0.69%, 95% CI −0.98 to −0.40; p = 0.0001). In the South, rates increased by 0.88% per year (0.53-1.24) until 2017 and declined by 2.25% per year (−3.08 to −1.41) thereafter; the overall trend was stable (AAPC −0.04%, −0.28 to 0.20). In the Central-West, mortality increased by 2.83% per year (1.95-3.72) until 2016 and declined by 2.64% per year (−4.10 to −1.16) thereafter, although the full-period AAPC remained positive at 0.97% (0.46-1.49). The North and Northeast had AAPCs of 5.23% (3.76-6.73) and 3.92% (3.30-4.55), respectively.

As a secondary, exploratory analysis, the age-adjusted interaction model confirmed regional heterogeneity (Wald χ² = 246.2, 4 df; p < 0.0001). Mortality declined in the Southeast by 1.03% per year (95% CI −1.32 to −0.74), increased in the North by 3.06% (2.05-4.08) and Northeast by 3.00% (2.52-3.47), remained stable in the South (−0.27%, −0.59 to 0.04), and increased modestly in the Central-West (0.71%, 0.05-1.36). The North recorded only seven melanoma deaths in 2000; the very low initial counts in the North and Northeast warrant caution because incomplete mortality ascertainment may have contributed to the steep early increases.

Sex-specific trends

**Figure 3:**
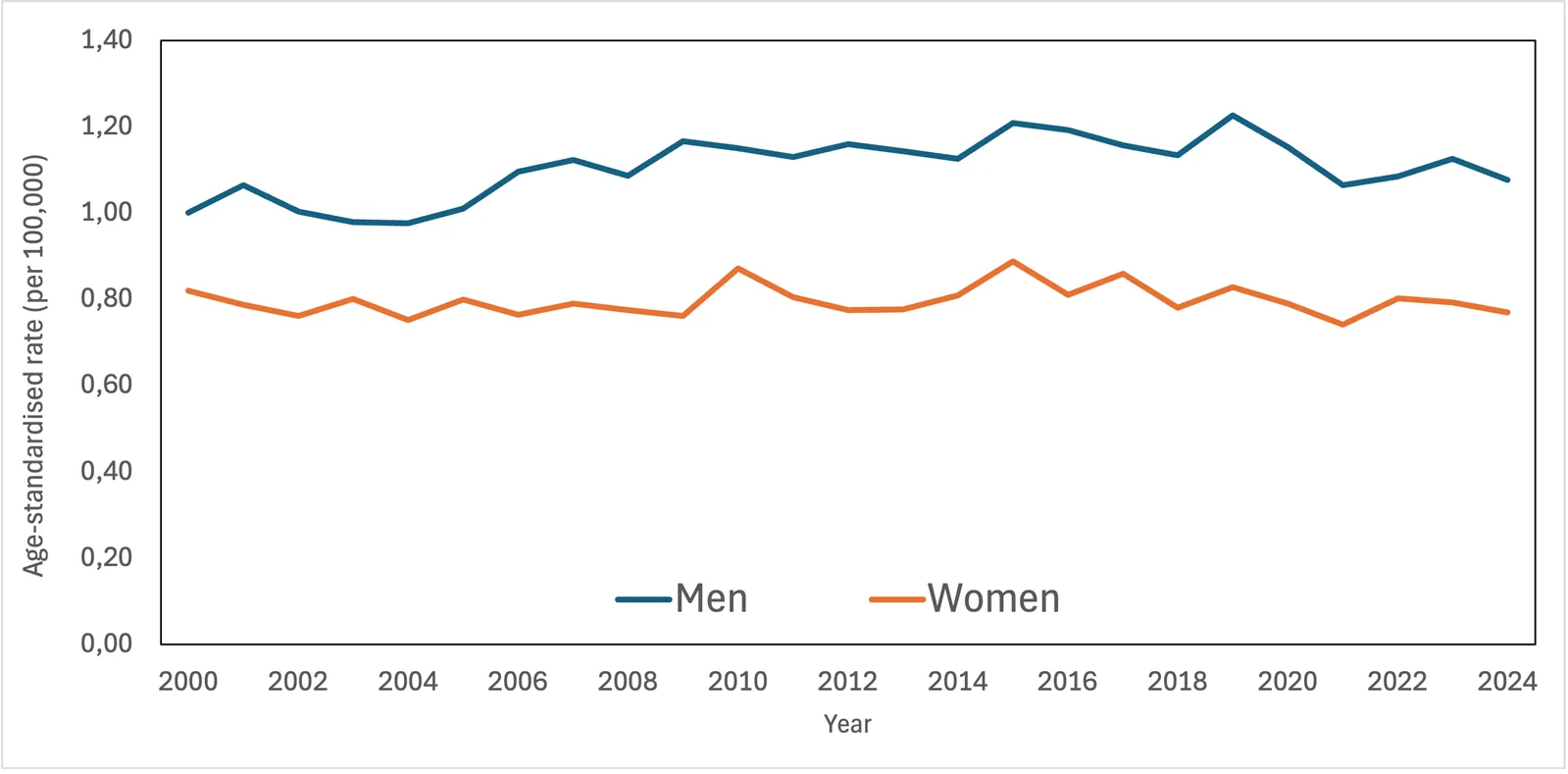
Age-standardized mortality rate for melanoma by sex in Brazil (2000-2024) Solid lines show the observed annual mortality rates for men and women. Sex-specific temporal patterns were estimated separately using segmented log-linear regression. Among men, mortality increased until 2015 and declined thereafter; among women, no joinpoint was identified, and the overall trend was stable.

Among men, mortality increased by 1.25% per year (95% CI 0.86-1.65) until 2015 and declined by 1.07% per year (−1.66 to −0.49) thereafter, yielding an AAPC of 0.38% (0.16-0.59) (Figure 3). No joinpoint was identified among women, whose full-period trend was stable (AAPC 0.03%, −0.24 to 0.31).

In the secondary age-adjusted negative binomial model, mortality declined by 0.43% per year among women (95% CI −0.74 to −0.11) and was stable among men (0.03%, −0.26 to 0.32). The year-by-sex interaction was nominally significant (p = 0.037). Because this exploratory interaction was not adjusted for multiple testing and the independently fitted segmented models yielded different categorical labels, the finding is interpreted as limited evidence of a possible difference in trajectory rather than as definitive evidence of a widening sex gap.

Age-specific trends

**Figure 4:**
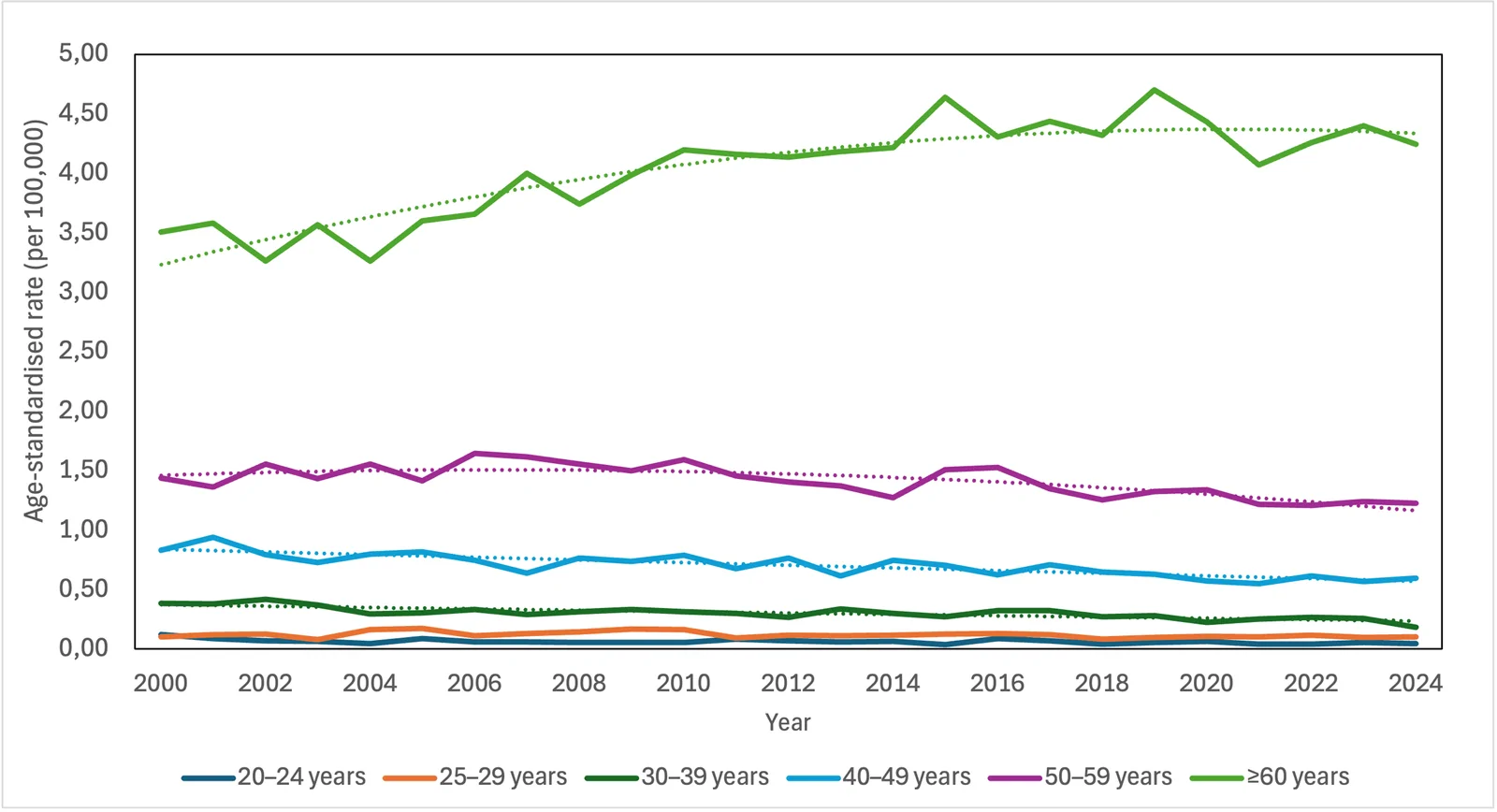
Age-specific mortality rate for melanoma by age group in Brazil for both sexes (2000-2024) Deaths were obtained from the Mortality Information System and population denominators from the 2024 revision of the IBGE projections, rebased to the 2022 Census. Lines show annual age-specific mortality rates within each age group and were not age-standardized. Dashed lines show fitted segmented log-linear trends, and red markers identify statistically selected joinpoints. Mortality declined throughout the study period in groups younger than 50 years, changed from an increase to a decline in adults aged 50-59 years in 2007, and changed from an increase to a stable trend among adults aged 60 years or older in 2015. IBGE: Brazilian Institute of Geography and Statistics

Mortality declined significantly in all age groups younger than 50 years, with no joinpoints identified (Figure 4). AAPCs were −2.38% (95% CI −4.44 to −0.27) at 15-19 years, −2.27% (−3.66 to −0.86) at 20-24 years, −1.25% (−2.41 to −0.08) at 25-29 years, −1.83% (−2.42 to −1.23) at 30-39 years, and −1.59% (−2.01 to −1.16) at 40-49 years.

Among adults aged 50-59 years, mortality increased by 1.64% per year (95% CI 0.18-3.12) until 2007 and declined by 1.58% per year (−2.02 to −1.12) thereafter, resulting in an AAPC of −0.65% (−1.02 to −0.28). Adults aged 60 years or older were the only group with an overall increase (AAPC 1.06%, 0.82-1.31). Their rate increased by 1.97% per year (1.52-2.43) until 2015 and was stable thereafter (−0.44%, −1.08 to 0.21; p = 0.173). The year-by-age-group interaction was significant (Wald χ² = 301.6, 7 df; p < 0.0001).

COVID-19 period

In the interrupted time-series analysis, mortality increased by 0.69% per year before 2020 (95% CI 0.44-0.94; p < 0.0001). The onset of the COVID-19 pandemic was associated with an immediate 6.60% reduction in mortality (p = 0.044), without a significant change in the subsequent slope (p = 0.216). The estimated post-2020 trend was −0.43% per year (−2.11 to 1.28). The segmented model identified the principal change in trajectory in 2016, four years before the pandemic. Model fit was similar for the segmented and interrupted time-series specifications (AIC 12.3 and 13.4, respectively).

Sensitivity analyses

The principal national findings were robust to the exclusion of 2024 and of the pandemic years. Excluding 2024 left the joinpoint in 2016 and the terminal APC at −1.10% per year (p = 0.004). After exclusion of 2020-22, the joinpoint again remained in 2016, and the terminal APC was −0.94% per year (p = 0.004).

Choice of population denominator vintage materially altered both the magnitude and direction of the full-period trend estimate: the national AAPC reversed from -0.39%/year (-0.60 to -0.18) to +0.17%/year (-0.01 to +0.36) after rebasing to the 2022 Census, with the same sign reversal observed in men and women (Table 2).

**Table 2:** Prespecified sensitivity analyses of the temporal trend in national age-standardized melanoma mortality in Brazil (2000-2024) Values are the estimated annual percentage changes in the age-standardized mortality rate, obtained using weighted log-linear regression on ln(rate), with weight = 1/Var[ln(rate)], and are presented with 95% CIs unless otherwise noted. S1 and S2 refit the primary segmented model with the joinpoint fixed at 2016, excluding the indicated year(s) by setting their regression weight to zero. S3 repeats the full analysis using the population denominator series prior to rebasing by the 2022 Census. S4 fits separate, unsegmented trends before and after 2020. All estimates use the rebased (2022 Census) population series unless otherwise specified. AAPC: average annual percent change, APC: annual percent change, CI: confidence interval

Sensitivity analysis	Estimate (%/year)	95% CI	Comparison with primary analysis
S1 - exclusion of the final year (2024)	-1.10	-1.80 to -0.40	Primary, 2016-2024 (terminal segment): -1.12 (-1.69 to -0.54)
S2 - exclusion of pandemic years (2020-2022)	-0.94	-1.55 to -0.34	Primary, 2016-2024 (terminal segment): -1.12 (-1.69 to -0.54)
S3 - pre-2022-Census population series (national)	-0.39	-0.60 to -0.18	Primary, rebased 2022 Census (AAPC 2000-2024): +0.17 (-0.01 to +0.36)
S4 - separate linear trends, 2000–2019 vs. 2020–2024	Pre-2020: +0.69	+0.44 to +0.94	Primary, single trend 2000–2024 (AAPC): +0.17 (-0.01 to +0.36)
	Post-2020: -0.43	-2.11 to +1.28	

When separate linear trends were fitted before and after 2020, mortality increased by 0.69% per year during 2000-2019 (95% CI 0.44-0.94; p < 0.0001) and showed no significant trend during 2020-2024 (−0.43%, 95% CI −2.11 to 1.28).

## Discussion

In this nationwide analysis of cutaneous melanoma mortality in Brazil, the age-standardized rate rose modestly until 2016 and declined thereafter, netting out to a stable trend over the full 25-year period even as annual deaths nearly doubled. Men had higher melanoma mortality than women throughout the study period, although evidence regarding whether this gap widened differed between modeling approaches: sex-specific segmented models and the age-adjusted interaction model yielded somewhat different patterns. Mortality declined in every age group under 60 years and rose only among the oldest adults, a rise that had flattened by 2015. Regional trajectories diverged sharply, from a sustained Southeast decline to rising rates from a low base in the North and Northeast. The COVID-19 pandemic was associated with a transient reduction in recorded deaths without altering the underlying temporal slope. Furthermore, revising the population denominators to incorporate the 2022 Census, rather than any biological signal, changed the national trend estimate from a statistically significant decline to a statistically nonsignificant overall trend, a finding we think matters well beyond this one disease.

The effect of population-denominator revision warrants particular attention because it substantially altered the interpretation of the overall temporal trend. Annual deaths climbed from 1,027 to 2,031, while the age-standardized rate remained essentially unchanged, meaning population growth and aging, not a rising risk of dying from melanoma, drove almost all of the added burden; crude counts still matter for service planning but should not be read as evidence that individual risk doubled. More striking was how much the denominator itself mattered: swapping the pre-Census population series for the rebased 2024 IBGE projections moved the national AAPC from a significant −0.39% per year to a nonsignificant 0.17%, with a comparable shift in the sex-specific estimates. Denominators are usually treated as fixed scaffolding in mortality studies; our findings demonstrate that major revisions to population denominators can materially affect whether a temporal trend is classified as increasing, decreasing, or stable, a caution that should generalize to any country fitting joinpoint or Poisson models across a Census update.

Read against the existing Brazilian literature, our segmented curve helps reconcile results that looked contradictory. Mendes et al. described rising mortality between 1980 and 2005, especially among older adults [16]; Santos and Souza then found a national decline through 2012, with marked regional variation [9]; de Melo et al. saw a modest rise in men and stability in women through 2014 [17], and a São Paulo registry reported stable overall mortality with increases concentrated in men and adults aged 60 years or older [18]. These apparently divergent findings may reflect different observation windows within the same nonlinear temporal trajectory: an early increase, a prolonged plateau, and a more recent decline that could be identified only with extended follow-up.

Age was the clearest source of heterogeneity in the observed mortality trends. Mortality fell in every group under 50 and, after 2007, in the 50-59 group as well; only adults aged 60 years or older showed an overall increase in mortality, consistent with earlier Brazilian work identifying this group as persistently disadvantaged [18,19], though our results suggest the disadvantage is no longer accelerating. Death records alone cannot say whether this reflects cumulative ultraviolet exposure, later-stage diagnosis, comorbidity, or a cohort effect.

The sex-specific findings warrant cautious interpretation, since our two approaches agreed that a sex difference existed but not on its temporal trajectory. Men had the higher rate throughout, in keeping with international evidence of later-stage presentation and worse anatomically adjusted survival [3,4]. But whereas the segmented models showed men rising then falling and women holding flat, the age-adjusted interaction model pointed to a small decline among women against stability in men, a secondary, exploratory comparison, unadjusted for multiple testing and only just below conventional significance. We regard the evidence for a divergence in trajectory as limited and inconsistent across modeling approaches, and a more robust conclusion is that the male excess persisted throughout the study period.

Brazil's post-2016 decline fits a broader pattern of falling mortality alongside rising or plateauing incidence elsewhere [2,20], usually attributed to earlier detection and better systemic treatment; in the USA, mortality fell by more than 6% a year between 2013 and 2017 after checkpoint inhibitors and BRAF-targeted therapy reached practice [21]. Brazil's decline was real but considerably smaller, and our data cannot apportion it between diagnosis, stage migration, and treatment access.

Marked geographic heterogeneity was also observed. The South remained the highest-mortality region throughout, consistent with its larger population of European descent and lighter constitutive phototype [5,6] and with previously mapped clusters in Rio Grande do Sul, Santa Catarina, and Paraná [22], yet it too turned downward after 2017. The Southeast showed the most consistent improvement, extending earlier reports from that region [19]. The North and Northeast posted the steepest rises, from just seven deaths in the North in 2000, a count so small that improving death ascertainment almost certainly inflated the early slope; given these small initial counts, the magnitude of the increase should be interpreted cautiously; the broader regional pattern is more informative: decline in the Southeast, stability in the South, and a less favorable trajectory in the North and Northeast.

The effect associated with the pandemic was limited. An immediate 6.6% drop in recorded deaths in 2020, against Brazilian hospital data showing falling admissions for skin malignancies [10] and international reports of delayed melanoma diagnoses [11], may reflect disruptions in care-seeking and death certification rather than a biological effect, since a true treatment gap would show up as a later slope change, and none appeared. The national joinpoint remained at 2016 whether or not the pandemic years were included, indicating that the downturn preceded the COVID-19 pandemic.

Our analysis carries the usual limits of ecological, registry-based work: no individual-level data on stage, Breslow thickness, phototype, ethnicity, or treatment; and death-certification quality that plausibly varies by region and era, bearing most heavily on our earliest North and Northeast estimates. In particular, the absence of stage-specific mortality and treatment-related data (systemic therapy, surgery, or time to treatment) means we cannot determine what fraction of the post-2016 decline reflects earlier detection versus improved access to systemic therapy. Standardizing to the 2022 Brazilian population sharpens internal comparisons at the cost of comparability with studies using world or European standards, and even our preferred, rebased denominator series remains a modeled estimate, not a Census count in every year. One data-entry error affecting Southeast counts was caught during validation and corrected, with every affected analysis rerun.

## Conclusions

Brazil has entered a more favorable recent phase of melanoma mortality, but the transition remains incomplete and geographically unequal. Age-standardized mortality has declined since 2016 despite continued growth in absolute deaths. Men remain at higher risk, younger and middle-aged adults have benefited most, and the most unfavorable mortality trajectories are increasingly observed in the North and Northeast. In contrast, rates have stabilized or declined in the historically higher-burden South and Southeast. Taken together, these findings suggest that melanoma mortality in Brazil is no longer defined primarily by the regions with the highest historical burden but increasingly by those undergoing the fastest epidemiological change. Future control strategies should move beyond uniform national policies and incorporate region-specific approaches that acknowledge the country’s marked demographic, phenotypic, and healthcare heterogeneity.
